# Asthma occurrence in children and early life systemic antibiotic use: an incidence density study

**DOI:** 10.1186/s13223-023-00773-8

**Published:** 2023-03-06

**Authors:** Hayat Bentouhami, Milcah Kahkelam Bungwa, Lidia Casas, Samuel Coenen, Joost Weyler

**Affiliations:** 1 Department of Family Medicine & Population Health (FAMPOP), Social Epidemiology and Health Policy (SEHPO), University of Antwerp, Antwerp, Belgium; 2 Department of Family Medicine & Population Health (FAMPOP), Primary Care & Interdisciplinary Care Antwerp (ELIZA), University of Antwerp, Antwerp, Belgium; 3 Laboratory of Medical Microbiology, Vaccine & Infectious Disease Institute (VAXINFECTIO), University of Antwerp, Antwerp, Belgium; 4 StatUa Statistics Centre, University of Antwerp, Antwerp, Belgium

**Keywords:** Asthma, Antibiotics, Incidence density study, Respiratory tract infections

## Abstract

**Background:**

Results of studies evaluating the relationship between asthma occurrence and early life antibiotic use have been conflicting. The aim of this study was to investigate the relationship between occurrence of asthma in children and systemic antibiotic use in the first year of life based on an incidence density study with careful consideration of the temporal aspects of the determinant-outcome relationship.

**Methods:**

We conducted an incidence density study nested in a data collection project with information on 1128 mother–child pairs. Systemic antibiotic use in the first year of life was defined as excessive (≥ 4 courses) vs. non-excessive (< 4 courses) use based on information from weekly diaries. Events (cases) were defined as the first parent-reported occurrence of asthma in a child between 1 and 10 years of age. Population time ‘at risk’ was probed by sampling population moments (controls). Missing data were imputed. Multiple logistic regression was used to assess the association between current first asthma occurrence (incidence density) and systemic antibiotic use in the first year of life, to evaluate effect modification and adjust for confounding.

**Results:**

Forty-seven first asthma events and 147 population moments were included. Excessive systemic antibiotic use in the first year of life showed more than twice the incidence density of asthma compared to non-excessive use (adjusted IDR [95% CI]: 2.18 [0.98, 4.87], p = 0.06). The association was more pronounced in children who have had lower respiratory tract infections (LRTIs) in the first year of life compared to children who had no LRTIs in the first year of life (adjusted IDR [95% CI]: 5.17 [1.19, 22.52] versus 1.49 [0.54, 4.14]).

**Conclusions:**

Excessive use of systemic antibiotics in the first year of life may play a role in the genesis of asthma in children. This effect is modified by the occurrence of LRTIs in the first year of life, with a stronger association observed in children experiencing LRTIs in the first year of life.

**Supplementary Information:**

The online version contains supplementary material available at 10.1186/s13223-023-00773-8.

## Background

Studies worldwide have found disparities in the prevalence of asthma between countries [1]. It has been suggested that these disparities are more likely explained by environmental exposures than by genetic differences [2]. However, there is still a poor understanding of the mechanisms through which factors that are considered fundamental to the development of asthma act. It is now commonly accepted that asthma develops from the complex interplay between genetic and environmental factors [3, 4]. In order to explain the marked differences in the prevalence of asthma between countries, some interesting hypotheses have been proposed. One of the first commonly accepted is the hygiene hypothesis. This hypothesis states that the increase in the occurrence of allergic illnesses (including asthma), can be explained by changes in living conditions leading to a reduction in exposure to microorganisms and infections in early life [5, 6]. As antibiotics act as an antimicrobial agent and might play a role in different pathways underlying the hygiene hypothesis, several studies have investigated the potential role of antibiotic use in the occurrence of asthma [7].

Currently, three pathways through which antibiotics may have an influence on the prevalence of allergic illnesses (including asthma) have been proposed. Firstly, antibiotics may remove the protective effect of bacterial infections that are hypothesized to protect against allergic illnesses by modifying the natural course of these infections [5, 7]. Secondly, antibiotics with potential anti-inflammatory effects, such as macrolides, may directly inhibit the type 1 immunity response and trigger the development of allergic illnesses [7, 8]. Thirdly, antibiotics may act through their influence on the gut microbiome [8–10]. Animal studies have shown that perinatal exposure to antibiotics impacts the gut microbiome and increases the susceptibility to allergic illnesses through the Th2 model [9]. In some epidemiological studies a dose–effect relationship between asthma occurrence and antibiotic use in the first year of life has been observed [11, 12]. In two studies the highest risk for asthma was observed in children receiving four or more courses of antibiotics [12, 13]. It has been advocated that excessive antibiotic consumption should be avoided given the potential induction of microbial resistance and the possible association with health outcomes such as asthma [3, 7, 14]. However, in several European countries antibiotics are still overprescribed and trends for the consumption of antibiotics in Europe hardly changed since 1997 [15, 16].

The true nature of the association between asthma occurrence and antibiotic use however remains unclear. Some studies demonstrated a positive association between antibiotic use in early life and the occurrence of asthma [17–19], whereas others failed to demonstrate an association [20–23]. It has been investigated whether these conflicting results could be explained by ‘protopathic bias’ or ‘reversed causation’ [22, 24–26]. To allow for a causal interpretation on a determinant(s)-outcome relationship in etiological research, it is quintessential that the temporal aspects of the determinant(s)-outcome relationship are carefully included in the study design (theoretical design, design of data collection and design of data processing) [27]. Consequently, the aim of this study was to investigate the relationship between current occurrence of asthma in children and antecedents of systemic antibiotic use in the first year of life with careful consideration of the temporal aspects of the determinant-outcome relationship.

## Methods

### Theoretical design

To answer the research question ‘what is the relationship between asthma occurrence in children and antecedents of systemic antibiotic use in the first year of life?’ this study aims to estimate current incidence density of first asthma occurrence as a function of antecedents of systemic antibiotic use in the first year of life in the domain ‘children’ (i.e. persons between birth and puberty). The ‘primary endpoint’ of this study is the incidence density ratio (IDR) as a measure of the strength of the association. In order to allow for a causal interpretation, sex, parental education, breast feeding for at least 6 months, lower respiratory tract infections (LRTIs), paracetamol (acetaminophen) use in the first year of life, day-care attendance, environmental tobacco smoke (ETS), parental asthma and atopic dermatitis prior to onset were considered either as modifiers or confounders.

### Design of data collection

An incidence density study was set up [28] nested within a data collection project collecting information on 1128 mother–child pairs. The project, the Prospective Study on the Influence of Perinatal factors on the Occurrence of Asthma and Allergies (PIPO), started in the province of Antwerp (Belgium) in 1997 [29, 30]. In the project 2000 pregnant women were invited to participate in order to include a sufficient number of children in the study (aimed sample size was 1200).

Consequently, an appropriate number of respiratory health outcomes would become available during the observation period for precise parameter estimation in studies assessing the relationship between perinatal exposures and respiratory health outcomes.

Data on demographic characteristics, health status, lifestyle and environmental exposures of the mothers and children were collected during two home visits; one at 5 months of pregnancy and one at 3 months post-partum, bi-annually between birth and 4 years of age and annually between 4 and 10 years of age. The questionnaires were based on the standardized questionnaires of the International Study of Asthma and Allergies in Childhood (ISAAC) [31]. However, for asthma definition we did not use the ISAAC questions. During the first year of life, a diary was kept by the parents for weekly registration of respiratory symptoms and names of medications administered to the infant.

#### Sampling

As current incidence density cannot be assessed directly and our aim was to only look at relative current incidence density (IDR), we decided to sample according to the principles of a case–control study redefined as explained by Miettinen [28]. In this sampling approach, cases are included as events whereas controls (population moments) are included for probing population time ‘at risk’ [28]. We decided to assess current incidence density of a (first) parent-reported asthma occurrence in an observation period of 9 years (between 1 and 10 years of age) in order to sample within the domain of the study and in order to collect a sufficient number of events.

#### Events (first asthma occurrence):

Information on asthma occurrence was parent-reported and obtained from the bi-annual and annual questionnaires. First asthma occurrence was defined as answering for the first time ‘yes’ to the question *“Has your child had asthma in the past 6 months* (between 1 and 4 years)*/12 months* (between 4 and 10 years)*?”*.First asthma events under the age of 1 year were not included, since the exposure status could only be completed at 1 year of age (cf. infra). Inclusion of first asthma events under the age of 1 year would also not allow the assessment of exposure prior to occurrence of the first asthma event, because no information on the timing of diagnosis was available. Also, diagnosis of asthma under the age of 1 year is even more complex than at other ages [32, 33]. Since the domain of the study is children, and puberty can already start at 10 years, [34] all events occurring between 1 and 10 years of age were included in the study.

#### Population moments

Eligible population moments (controls) were children still at risk for developing the event (at each follow-up within the PIPO project). Sampling of population moments was performed within the same observation period as the period for collecting the events (i.e. between 1 and 10 years of age). This was completed in two stages, first by pooling all eligible population moments in a ‘risk set’. This ‘risk set’ then contained all population moments still ‘at risk’ for the event within the observation period. Secondly, a three times larger (than the events) sample of population moments was randomly (and unmatched) taken from the ‘risk set’.

### Exposure to systemic antibiotics

Information on systemic antibiotic use (including type of antibiotic) in the first year of life was obtained from the weekly diaries. For all events and population moments, the number (courses) of systemic antibiotics (administered either orally, intravenously or intramuscularly) weekly was counted. Systemic antibiotic use in the first year of life was operationalized in two categories: excessive use (≥ 4 courses) and non-excessive use (< 4 courses). As the duration of a course is more or less one week, receiving one course for at least four weeks was considered as excessive use as well. When no information on the exposure was provided in a certain week, this week was considered as a ‘missing week’. In case of missing weeks, the number of missing weeks was assessed and the potential impact on the classification in excessive or non-excessive use of systemic antibiotics was evaluated. When the missing weeks might have led to misclassification for the exposure, the questionnaire at 1 year of age was consulted. In case of discordant information, the event or population moment was excluded from the study.

### Relevant characteristics

Information on relevant characteristics was obtained from the mother’s and father’s questionnaires at inclusion, during pregnancy, immediately after birth and the bi-annual and annual questionnaires. The procedure differed between events and population moments.

For both events and population moments, information on sex (biological), parental education and breast feeding for at least 6 months was obtained from the questionnaires in the first year of life. Information on parental asthma was obtained from the mother’s and father’s questionnaire. Information on LRTIs (defined as having had bronchitis with or without chronic cough and/or pneumonia according to the reporting of the parents) and paracetamol (acetaminophen) use in the first year of life was obtained from the questionnaire at 1 year of age. Information on day-care attendance and atopic dermatitis was obtained from all questionnaires (3 months post-partum, bi-annual and annual) prior to or at onset (for events) or prior to or at sampling (for population moments). Information on ETS, which is a time-varying characteristic, was obtained from all questionnaires (at 3 months post-partum, bi-annual and annual) prior to or at first asthma diagnosis (for events) or at sampling (for population moments).

#### Design of data processing

Missing data were imputed by applying Multiple Imputation by Chained Equations (MICE) [35]. A more detailed explanation of this procedure can be consulted in the Additional file 1.

Crude incidence density ratio (IDR) was calculated based on the odds ratio in a 2 × 2-table including exposure states in first asthma events and population moments and for inference a 95% confidence interval (CI) was estimated [28]. Interaction terms between the exposure and potential effect modifiers were included in multiple logistic regression models to assess effect modification. For deciding on the inclusion or exclusion of an interaction term, an α-level of 0.20 was used [36]. Adjusted IDRs and 95% CIs were estimated by multiple logistic regression accounting for potential modifiers and adjusting for (actual) confounders. Additionally, all statistical modelling was also performed in the complete cases (without imputation of missing data) and can be consulted in the supplementary material (Additional file 2).

All relevant statistical information from the output of the regression models (regression coefficients, standard errors, IDRs, 95% CIs and p-values) is presented to facilitate the interpretation of the models [37].

Statistical modelling was carried out in R version 4.2.2 [38].

## Results

### Sampling

**Fig. 1:**
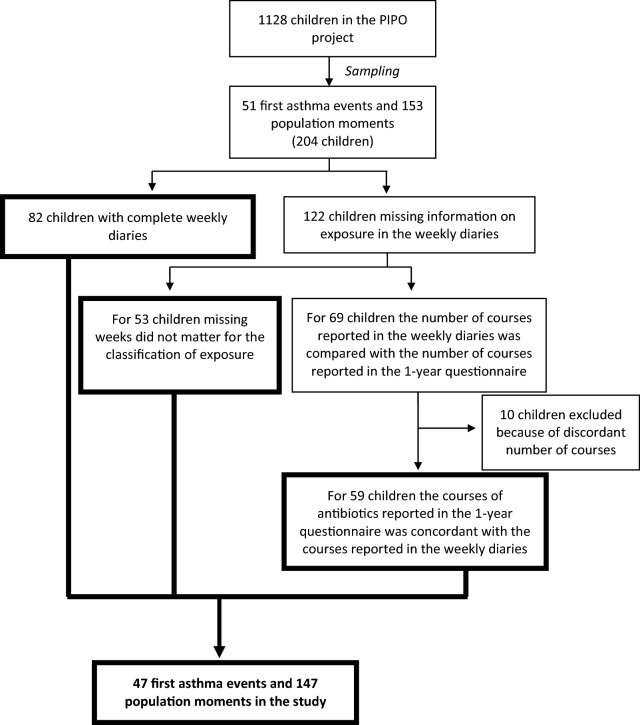
Selection of first asthma events and population moments (controls) taking into account missing exposure in weekly diaries

Within the data collection project with information on 1128 mother–child pairs, 51 first asthma events were identified and 153 population moments were randomly (i.e. not matched) sampled from the ‘risk set’. From all 204 records (first asthma events and population moments), 82 (42.3%) had complete weekly diaries (one complete weekly diary equals 52 weeks of information) and 122 (57.7%) had at least 1 missing week with respect to antibiotic use. The missing weeks and the possible impact on the classification for the exposure was carefully inspected (Fig. 1). Forty-seven first asthma events and 147 population moments remained for inclusion in the study.

### Relevant characteristics

Table 1 shows the characteristics of the children in whom first asthma events (n = 47) were sampled from (‘the events’) and in whom population time (n = 147) was sampled from (‘the population moments’). The number of courses of systemic antibiotics received in the first year of life ranged from 0 to 13, both in the events and in the population moments.

**Table 1:** Characteristics of the children in whom the events and population time were sampled from

	First asthma events (n = 47)	Population moments (n = 147)
Age, months, median (min; max)	48 (18; 108)	42 (18; 108)
Sex, female, n (%)	15 (31.9)	70 (47.6)
Parental education, high, n (%)	38 (86.4)	122 (88.4)
Day-care attendance, yes, n (%)	33 (82.5)	118 (82.5)
ETS, yes, n (%)	7 (20.6)	10 (6.8)
Breastfed for at least 6 months, yes, n (%)	10 (22.7)	46 (31.7)
LRTIs first year of life, yes, n (%)	12 (26.1)	39 (26.7)
Paracetamol (acetaminophen) use first year of life, yes, n (%)	40 (87.0)	115 (82.1)
Parental asthma, yes, n (%)	9 (20.5)	22 (15.9)
Atopic dermatitis, yes, n (%)	31 (70.5)	61 (44.9)
Number of systemic antibiotic courses in the first year of life, n (%)		
0	20 (42.6)	69 (46.9)
1	6 (12.8)	21 (14.3)
2	4 (8.5)	17 (11.6)
3	3 (6.4)	12 (8.2)
4	3 (6.4)	7 (4.8)
5	3 (6.4)	3 (2.0)
6	3 (6.4)	6 (4.1)
7	1 (2.1)	7 (4.8)
8	1 (2.1)	2 (1.4)
9	1 (2.1)	2 (1.4)
11	1 (2.1)	0 (0.0)
13	1 (2.1)	1 (0.7)
Courses systemic antibiotics in the first year of life		
Excessive (≥ 4 courses)*	14 (29.8)	28 (19.0)
Non-excessive (< 4 courses)	33 (70.2)	119 (80.9)

*ETS* Environmental tobacco smoke; *LRTIs* Lower respiratory tract infections; * IDR 1.80 (95% CI 0.85, 3.81), *chi*^*2*^ 2.42, p = 0.12

### Missing data on relevant characteristics

There were no missing data for (first) asthma occurrence, exposure and sex. In total, 57 (29.4%) children in the study had missing data for at least one of the remaining characteristics of interest (17 first asthma events and 40 population moments).

### Association between (first) asthma occurrence and antecedents of systemic antibiotic use in the first year of life

#### Crude incidence density ratio (IDR)

In the first asthma events, 14 (29.8%) received four or more courses of systemic antibiotics in the first year of life, whereas in the population moments 28 (19.0%) received four or more courses of systemic antibiotics in the first year of life (Table 1). The odds ratio as an estimator for the crude IDR comparing ‘excessive exposed’ with ‘non-excessive exposed’ was 1.80 (95% CI 0.85, 3.81).

#### Statistical modelling

The main findings from the multiple logistic regression models using the imputed datasets are presented in Table 2. The strength of the association differed with LRTIs in the first year of life, sex, parental education, ETS, parental asthma and atopic dermatitis, but only for ‘LRTIs in the first year of life’ the estimation of the regression coefficient of the interaction term was precise enough (p-value of the interaction term below the α-level of 0.20) to consider modification. The association between current first asthma occurrence and systemic antibiotic use in the first year of life was more pronounced in children with reported LRTIs in the first year of life (IDR [95% CI] 5.17 [1.19, 22.52]) compared to children with no reported LRTIs in the first year of life (IDR [95% CI] 1.49 [0.54, 4.14]).

**Table 2:** Results based on crude and adjusted models after multiple imputation of missing data for the association between first asthma occurrence in children and excessive systemic antibiotic use (≥ 4 courses) in the first year of life

	Β	SE	IDR	95% CI	p
Crude model					
Excessive systemic antibiotic use in the first year of life	0.59	0.38	1.80	(0.85, 3.81)	0.12
Adjusted models					
Excessive systemic antibiotic use^a^	0.78	0.41	2.18	(0.98, 4.87)	0.06
Evaluation of effect modification by sex					
Crude model					
Excessive systemic antibiotic use for sex = male	0.26	0.46	1.29	(0.52, 3.20)	0.57
Excessive systemic antibiotic use for sex = female	1.04	0.69	2.82	(0.72, 10.99)	0.14
Interaction term	0.78	0.83	–	–	0.35
Adjusted model^b^					
Excessive systemic antibiotic use for sex = male	0.44	0.48	1.56	(0.60, 4.03)	0.36
Excessive systemic antibiotic use for sex = female	1.25	0.77	3.49	(0.77, 15.72)	0.10
Interaction term	0.81	0.90	–	–	0.37
Evaluation of effect modification by parental education					
Crude model					
Excessive systemic antibiotic use for parental education = low	0.77	0.97	2.16	(0.32, 14.37)	0.43
Excessive systemic antibiotic use for parental education = high	0.56	0.42	1.75	(0.76, 4.01)	0.19
Interaction term	− 0.21	1.06	–	–	0.84
Adjusted model^c^					
Excessive systemic antibiotic use for parental education = low	1.38	1.11	3.99	(0.45, 35.42)	0.21
Excessive systemic antibiotic use for parental education = high	0.69	0.44	1.99	(0.84, 4.72)	0.12
Interaction term	− 0.70	1.19	–	–	0.56
Evaluation of effect modification by ETS					
Crude model					
Excessive systemic antibiotic use for ETS = no	0.60	0.43	1.83	(0.78, 4.28)	0.16
Excessive systemic antibiotic use for ETS = yes	1.19	1.26	3.29	(0.28, 38.78)	0.35
Interaction term	0.58	1.34	–	–	0.66
Adjusted model^d^					
Excessive systemic antibiotic use for ETS = no	0.69	0.44	1.99	(0.84, 4.73)	0.12
Excessive systemic antibiotic use for ETS = yes	1.53	1.30	4.62	(0.36, 59.46)	0.24
Interaction term	0.84	1.38	–	–	0.54
Evaluation of effect modification by LRTIs in the first year of life					
Crude model					
Excessive systemic antibiotic use for LRTIs first year of life = no	0.26	0.50	1.30	(0.49, 3.45)	0.60
Excessive systemic antibiotic use for LRTIs first year of life = yes	1.31	0.69	3.70	(0.96, 14.24)	0.06
Interaction term	1.05	0.85	–	–	0.22
Adjusted model^e^					
Excessive systemic antibiotic use for LRTIs first year of life = no	0.40	0.52	1.49	(0.54, 4.14)	0.44
Excessive systemic antibiotic use for LRTIs first year of life = yes	1.64	0.75	5.17	(1.19, 22.52)	0.03
Interaction term	1.24	0.91	–	–	0.17
Evaluation of effect modification by parental asthma					
Crude model					
Excessive systemic antibiotic use for parental asthma = no	0.53	0.41	1.69	(0.75, 3.81)	0.20
Excessive systemic antibiotic use for parental asthma = yes	1.74	1.29	5.71	(0.45, 72.29)	0.18
Interaction term	1.21	1.36	–	–	0.37
Adjusted model^f^					
Excessive systemic antibiotic use for parental asthma = no	0.78	0.45	2.18	(0.91, 5.27)	0.08
Excessive systemic antibiotic use for parental asthma = yes	2.14	1.38	8.54	(0.56, 128.95)	0.12
Interaction term	1.36	1.45	–	–	0.35
Evaluation of effect modification by atopic dermatitis					
Crude model					
Excessive systemic antibiotic use for atopic dermatitis = no	0.99	0.63	2.70	(0.78, 9.31)	0.12
Excessive systemic antibiotic use for atopic dermatitis = yes	0.42	0.51	1.52	(0.56, 4.12)	0.41
Interaction term	− 0.57	0.82	–	–	0.48
Adjusted model^g^					
Excessive systemic antibiotic use for atopic dermatitis = no	1.11	0.64	3.04	(0.86, 10.71)	0.09
Excessive systemic antibiotic use for atopic dermatitis = yes	0.64	0.55	1.89	(0.64, 5.54)	0.25
Interaction term	− 0.47	0.85	–	–	0.58

*ETS* Environmental tobacco smoke; *LRTIs* Lower respiratory tract infections; *β* regression coefficient; *SE* standard error; *IDR* incidence density ratio; *CI* confidence interval; *p* p-value

^a^Adjusted for confounding by parental education and ETS

^b^Adjusted for confounding by parental education and ETS and taking into account effect modification by sex

^c^Adjusted for confounding by ETS and taking into account effect modification by parental education

^d^Adjusted for confounding by parental education and taking into account effect modification by ETS

^e^Adjusted for confounding by parental education and ETS and taking into account effect modification by LRTIs in the first year of life

^f^Adjusted for confounding by parental education and ETS and taking into account effect modification by parental asthma

^g^Adjusted for confounding by parental education and ETS and taking into account effect modification by atopic dermatitis

In order to allow for comparison with the findings of other studies, the adjusted IDR without interaction term for LRTIs in the first year of life was estimated. Adjusting for confounding by parental education and ETS resulted in an IDR of 2.18 (95% CI 0.98, 4.87). Other characteristics (sex, day-care attendance, breast feeding for at least 6 months and paracetamol (acetaminophen) use in the first year of life) hardly confounded the association, with no changes in the regression coefficient more pronounced than 0.1 decimals.

## Discussion

Based on our findings and after adjustment for confounding by parental education and ETS, children exposed to four or more courses of systemic antibiotics in the first year of life seemed to have more than twice the incidence density for asthma occurrence than children exposed to less than four courses (IDR 2.18 (95% CI 0.98, 4.87)). Although our estimation was rather precise (p = 0.06) this association is not statistically significant at the predominantly used α-level of 0.05. Similar associations have been reported in previous studies. [39–41] Ni et al. reported in their study that antibiotic use in the first year of life is associated with asthma occurrence in children between 1 and 10 years of age (OR [95% CI] 2.66 [1.11, 6.40]) [40]. Su et al. reported in their study that use of systemic antibiotics in the first 9 months of life is associated with asthma in children up until the age of 5 years (adjusted OR: 1.50, p = 0.047). [42]

In the theoretical design, we indicated that the interest within this study was also to investigate possible effect modification by sex, parental education, ETS, LRTIs in the first year of life, parental asthma and atopic dermatitis. For the specified characteristics, interaction terms were added to the model. Deciding on whether or not to include the interaction term in the final model (and therefore whether or not to reject the null hypotheses of no interaction), was based on an α-level of 0.20. For sex, parental education, ETS, parental asthma and atopic dermatitis the interaction term exceeded the α-level of 0.20, limiting the evidence for effect modification. This was not the case for LRTIs in the first year of life. When including the interaction term for LRTIs in the first year of life in the adjusted model (where the association between excessive systemic antibiotic use and the occurrence of asthma was not significant at the α-level of 0.05), the adjusted association appeared to be much stronger in children who have had LRTIs in the first year of life, with a statistically significant IDR of 5.17 (p = 0.03) compared to in children who did not have LRTIs in the first year of life (IDR 1.49).

The observed association between excessive systemic antibiotic use in the first year of life and asthma occurrence in children who have had LRTIs in the first year of life, might be biologically explained by the composition of the lung microbiome [43, 44]. Both exposure to antibiotics and viral respiratory tract infections in early life can disrupt the composition of the lung microbiome, leading to an increased risk for the development of asthma [43, 45]. It is therefore plausible that the strong association observed in our study in children reporting LRTIs in the first year of life might be explained by a biological interplay between these two exposures. On the other hand children receiving an excessive amount of antibiotics in the first year of life and with LRTIs in the first year of life, might have received the antibiotics for the treatment of recurrent respiratory symptoms that are in fact not related to LRTIs. These recurrent respiratory symptoms in the first year of life might indeed already be an early sign of the presence of an obstructive pulmonary disease (asthma), implying that some children with this profile are predisposed for a later diagnosis of asthma (misclassification with respect to diagnosis of LRTIs). We assessed whether effect modification by LRTIs in the first year of life would still be observed after exclusion of the subjects reporting pneumonia in the first year of life. After exclusion of these subject (n = 9), effect modification by LRTIs was still observed. We therefore suggest that future studies with larger sample sizes elaborate on the possible biological pathways including the role of LRTIs.

Evidence for an association between exposure to antibiotics in early life and the occurrence of asthma has been controversial. Some studies failed to demonstrate an association between antibiotic use in early life and the occurrence of asthma in children [20]. These conflicting results could be (at least partially) explained by a mismatch between the ‘theoretical design’ of the study and the design of data collection. In etiological research, when the interest is in studying the *‘current occurrence of an event as a function of prior exposure’*, data on exposure and relevant characteristics (modifiers and confounders) must be collected prior to onset of the event or prior to and up to sampling as a population moment. When the interest is in studying the *‘future occurrence of an event as a function of current exposure’* (i.e. a prognosis oriented rather than an etiology oriented design), then data on relevant characteristics must be collected at the moment of the realization of the exposure. A mismatch between the theoretical design (etiology oriented, whether or not explicit) and the design of data collection (prognosis oriented), could therefore lead to misinterpretation of study results leading to conflicting study results [28]. In our study, the design of data collection was carefully set up to match with the ‘theoretical design’. Our theoretical design was etiology oriented (‘current occurrence of asthma as a function of past exposure to systemic antibiotics’). The design of data collection matched with the theoretical design, assessing current incidence density of asthma as a function of prior exposure to systemic antibiotics. All characteristics taken into account were prespecified in the theoretical design and temporality was also taken into account for these characteristics. We believe this is a strength of our study. [46, 47]

Another explanation for the diverging results in literature on the relationship between early life antibiotic use and the occurrence of asthma could be the population mix of the different studies and failing to take this population mix (regarding the proportion of children with LRTIs in the first year of life) into account. Depending on the prevalence of LRTIs in a study (high proportion of children with LRTIs vs. low proportion of children with LRTIs in the first year of life), it is more likely that the association between exposure to systemic antibiotics in the first year of life and occurrence of asthma would be observed in studies with a high proportion of children reporting LRTIs in the first year of life. Therefore, we advise further studies on the relationship between antibiotic use and occurrence of asthma in children to take LRTIs into account.

Our study has several weaknesses. The number of events in our study was low, which is reflected in the width of the confidence intervals for the association in children reporting LRTIs in the first year of life. Therefore, the results need to be interpreted with caution.

Asthma is a complex clinical disease difficult to diagnose under the age of 6 [48, 49]. According to a recent study by Yang et al., childhood asthma is often misdiagnosed [50]. In our study, 37 first asthma events under the age of 6 were included. This implies that some of the first asthma events could be misdiagnoses, but also that asthmatic children with respiratory symptoms in early life could have been missed and therefore included as population moments in our study. This problem of misclassification may have led to biased results. Moreover, if studies adjust for confounding by respiratory diagnoses (e.g. LRTIs) under the age of 6, overadjustment of the studied association might be a consequence if the respiratory diagnoses adjusted for are misdiagnoses. Being aware of this possibility of misclassification, we did include first asthma events under the age of 6 in our study, because we assumed that the antibiotic use in early life is probably more relevant for the events identified already in early life. For 13 out of the 37 first asthma events under the age of 6 the parents reported that the child suffered from asthma after the age of 6. We additionally assessed whether the other 24 first asthma events under the age of 6 reported other symptoms that could be typical for asthma. For 17 out of the 24 first asthma events wheezing and/or shortness of breath occurred at first asthma occurrence and was reported in at least one subsequent questionnaire. For the remaining 7 first asthma events, asthma was only reported in one questionnaire and symptoms were not persistent during the entire observation period, implying that these asthma events might be misclassified. We also checked for all asthma events whether asthma medication was used at the same time. For 40 out of 47 first asthma events, asthma medication was used at the moment of diagnosis. The other 7 first asthma events did not use asthma medication, however we decided to include them in our study because non-medicinal control of asthma is also possible (e.g. avoidance of exposures).

We did not look at antibiotic subtypes nor dose–effect, which might be important for the biological interpretation of our findings. Some studies investigated the relationship between exposure to subtypes of antibiotics and asthma in childhood. Örtqvist et al. reported in their study that the risk of asthma was more pronounced for exposure to antibiotics for the treatment of respiratory infections (HR [95% CI] 4.12 [3.78, 4.50]) compared to antibiotics for the treatment of urinary tract and skin infections (HR [95% CI] 1.54 [1.24, 1.92]) [51]. Unfortunately, the number of events in our study was too small to look at subtypes of antibiotics or dose–effect. We did not consider use of antibiotics during pregnancy, since the aim was limited to investigating the effect of exposure in the first year of life. Antibiotic use during childbirth was not considered, because no data on this exposure were available.

Despite the weaknesses, our study has several strengths. One of the strengths of our study is that the data used were collected in a project that was specifically designed to determine pre- and postnatal risk factors for childhood asthma. In the first year of life detailed information on antibiotic use was collected in weekly diaries. In most studies, such frequent collection of exposure information is rare and usually subjected to recall bias. In our study, the possibility of recall bias and misclassification for the exposure is minimized. Also, the collection of detailed information during the observation period allowed us to assess all relevant characteristics in accordance with the theoretical design of our study.

The probing of population time by (unmatched) sampling of population moments from a ‘risk set’ is an additional strength. If the population moments would have been sampled in the same databases the events were identified in, this would have led to matching on age which could have introduced bias [52]. Moreover, the procedure applied for sampling population moments allowed for including population moments in the study that were still ‘at risk’ for the event at the moment of sampling, but could have become an event later in time. Using this approach limits the risk for selection bias.

If the information on the exposure from the weekly diaries was inappropriate to allow for classification for the exposure status (as a consequence of missing weeks), the event or population moments was excluded from the study. Also, missing data were imputed using MICE. Most studies on this topic are often based on complete cases only. These results can be biased if selection bias is introduced because of missingness. We did also perform the statistical modelling with the complete cases only (see Additional file 2). The associations were stronger in the complete case analysis, but the confidence intervals were wider.

## Conclusion

In conclusion, the observed associations in our study indicate a relationship between the occurrence of parent-reported asthma in children and excessive systemic antibiotic use in the first year of life. The association was more pronounced (and statistically significant at the α-level of 0.05) in children who have had LRTIs in the first year of life. We therefore suggest to conduct further studies to assess in more detail the nature of this relationship between the occurrence of asthma and excessive systemic antibiotic use with special attention for the subtypes of antibiotics and dose–effect, focusing on the possible role of LRTIs in early life. Meta-analysis of existing studies or grouping data from different birth cohort projects could be a valuable basis for further research.

## Supplementary Information

**Additional file 1. **Design of data processing.

**Additional file 2. **Complete case approach.

## Data Availability

The data that support the findings of this study are available on request from the corresponding author. The data are not publicly available due to privacy or ethical restrictions.

## Abbreviations

CI: Confidence interval
ETS: Environmental tobacco smoke
IDR: Incidence density ratio
ISAAC: International Study of Asthma and Allergies in Childhood
LRTIs: Lower respiratory tract infections
MICE: Multiple imputation by chained equations
PIPO: Prospective study on the influence of perinatal factors on the occurrence of asthma and allergies
