# Supplementary material for: Asthma occurrence in children and early life systemic antibiotic use: an incidence density study
Source: Allergy Asthma Clin Immunol. 2023 Mar 6;19:18. doi: 10.1186/s13223-023-00773-8 (PMC9987135; doi:10.1186/s13223-023-00773-8)
Supplement: Supplementary file 1 — Additional file 1. Design of data processing. [file 13223_2023_773_MOESM1_ESM.docx]

**Design of data processing – additional information**

*Multiple imputation of missing data*

Missing data were imputed by applying Multiple Imputation by Chained Equations (MICE) under the assumption that missing data are missing at random (MAR).^35^ Data were not missing completely at random (MCAR), because missingness was associated with some of the characteristics of the population moments and the events. The number of imputations was arbitrarily set at 20 (29.4% of the children had a missing value for at least one of the included variables, but there was no difference between setting the number of imputations at 20 or more). In the imputation model, all variables of interest for the model with missing data (i.e.: parental education, breast feeding for at least 6 months, day-care attendance, LRTIs in the first year of life, paracetamol (acetaminophen) use in the first year of life, ETS, parental asthma and atopic dermatitis) were included. Additionally (to improve the precision of the imputation), auxiliary variables (variables not included in the model, but associated with missingness) were included in the imputation (i.e.: delivery method, the presence of older siblings, wheezing during the first year of life and exposure to pets). For imputation, the logistic regression method was used since all variables with missing data are binary variables.
