# Supplementary material for: Asthma occurrence in children and early life systemic antibiotic use: an incidence density study
Source: Allergy Asthma Clin Immunol. 2023 Mar 6;19:18. doi: 10.1186/s13223-023-00773-8 (PMC9987135; doi:10.1186/s13223-023-00773-8)
Supplement: Supplementary file 2 — Additional file 2. Complete case approach. [file 13223_2023_773_MOESM2_ESM.docx]

**Complete case approach**

**Association between (first) asthma occurrence and antecedents of exposure to systemic antibiotics in the first year of life: complete case approach (CCA)**

The main findings from the multiple logistic regression models in the CCA are presented in supplementary table 1. In the CCA, the crude odds ratio contrasting events and population moments as an estimator for the crude incidence density ratio (IDR) comparing ‘exposed’ with ‘unexposed’ was 2.24 (95% CI 0.93, 5.38), although not statistically significant at the α-level of 0.05. Possible effect modification by sex, parental education, ETS, LRTIs in the first year of life, parental asthma and atopic dermatitis was evaluated. The strength of the association differed with LRTIs in the first year of life, sex, parental education, ETS, parental asthma and atopic dermatitis but only for ‘LRTIs in the first year of life’ the estimation of the regression coefficient of the interaction term was precise enough (p-value of the interaction term below the α-level of 0.20) to consider modification. This suggests modification of the association between first parent-reported asthma occurrence and systemic antibiotic use in the first year of life by LRTIs in the first year of life. Current IDR comparing (first) asthma occurrence in children who received four or more courses of systemic antibiotics in the first year of life with (first) asthma occurrence in children who received less than four courses revealed a more pronounced (and statistically significant) effect in children with reported LRTIs in the first year of life compared to children with no reported LRTIs in the first year of life (IDR [95% CI]: 8.19 [1.38, 48.75], p = 0.04 versus 1.44 [0.42, 4.93], p = 0.55).

Adjusting for confounding by sex, parental education, day-care attendance and ETS, resulted in a more pronounced association (IDR [95% CI]: 2.52 [0.95, 6.63], p = 0.06). Although our estimation was rather precise, this association is not statistically significant at the predominantly used α-level of 0.05. Other characteristics (age, breastfeeding for at least 6 months and paracetamol (acetaminophen) use in the first year of life) hardly confounded the association, with no changes in the regression coefficient more pronounced than 0.1 decimals. In the final model in the CCA, both sex, parental education, day-care attendance and ETS confounded the association, whereas in the statistical modelling approach with the imputed datasets only parental education and ETS were identified as confounders of the association.

***Table S1:*** *Results based on crude and adjusted models in the complete case approach for the association between first asthma occurrence in children and excessive systemic antibiotic use (≥4 courses) in the first year of life*

|  | Β | SE | IDR | 95% CI | p | |
| --- | --- | --- | --- | --- | --- | --- |
| Crude model |  |  |  |  |  | |
| Excess systemic antibiotic use in the first year of life [events: n = 47; population moments: n = 147] | 0.59 | 0.38 | 1.80 | (0.85, 3.81) | 0.12 | |
| Excess systemic antibiotic use in the first year of life (CCA) [events: n = 30; population moments: n = 107] | 0.80 | 0.45 | 2.24 | (0.93, 5.38) | 0.07 | |
| Adjusted model^a^ |  |  |  |  |  | |
| Excess systemic antibiotic use in the first year of life | 0.92 | 0.49 | 2.52 | (0.95, 6.63) | 0.06 | |
| Evaluation of effect modification by sex |  |  |  |  |  | |
| Crude model |  |  |  |  |  | |
| Excess systemic antibiotic use for sex = Male | 0.47 | 0.56 | 1.59 | (0.53, 4.81) | 0.41 | |
| Excess systemic antibiotic use for sex = Female | 1.28 | 0.75 | 3.59 | (0.83, 15.54) | 0.09 | |
| Interaction term | 0.81 | 0.94 | - | - | 0.38 | |
| Adjusted model^b^ |  |  |  |  |  | |
| Excess systemic antibiotic use for sex = Male | 0.64 | 0.59 | 1.90 | (0.59, 6.09) | 0.28 | |
| Excess systemic antibiotic use for sex = Female | 1.49 | 0.82 | 4.43 | (0.89, 22.10) | 0.07 | |
| Interaction term | 0.85 | 0.98 | - | - | 0.39 | |
| Evaluation of effect modification by parental education |  |  |  |  |  | |
| Crude model |  |  |  |  |  | |
| Excess systemic antibiotic use for parental education = Low | 0.47 | 1.15 | 1.60 | (0.17, 15.27) | 0.68 | |
| Excess systemic antibiotic use for parental education = High | 0.87 | 0.49 | 2.40 | (0.91, 6.29) | 0.07 | |
| Interaction term | 0.40 | 1.25 | - | - | 0.75 | |
| Adjusted model^c^ |  |  |  |  |  | |
| Excess systemic antibiotic use for parental education = Low | 1.26 | 1.32 | 3.52 | (0.27, 46.44) | 0.34 | |
| Excess systemic antibiotic use for parental education = High | 0.88 | 0.52 | 2.40 | (0.86, 6.69) | 0.09 | |
| Interaction term | -0.38 | 1.38 | - | - | 0.78 | |
| Evaluation of effect modification by ETS |  |  |  |  |  | |
| Crude model |  |  |  |  |  | |
| Excess systemic antibiotic use for ETS = No | 0.74 | 0.48 | 2.09 | (0.81, 5.39) | 0.13 | |
| Excess systemic antibiotic use for ETS = Yes | 1.54 | 1.4 | 4.67 | (0.30, 73.38) | 0.27 | |
| Interaction term | 0.80 | 1.49 | - | - | 0.59 | |
| Adjusted model^d^ |  |  |  |  |  | |
| Excess systemic antibiotic use for ETS = No | 0.81 | 0.53 | 2.24 | (0.80, 6.27) | 0.12 | |
| Excess systemic antibiotic use for ETS = Yes | 1.84 | 1.45 | 6.29 | (0.37, 107.61) | 0.20 | |
| Interaction term | 1.03 | 1.52 | - | - | 0.50 | |
| Evaluation of effect modification by LRTIs in the first year of life | | | | | |  |
| Crude model |  |  |  |  |  | |
| Excess systemic antibiotic use for LRTIs first year of life = No | 0.36 | 0.59 | 1.43 | (0.45, 4.55) | 0.55 | |
| Excess systemic antibiotic use for LRTIs first year of life = Yes | 1.66 | 0.82 | 5.25 | (1.05, 26.20) | 0.04 | |
| Interaction term | 1.30 | 1.01 | - | - | 0.20 | |
| Adjusted model^e^ |  |  |  |  |  | |
| Excess systemic antibiotic use for LRTIs first year of life = No | 0.37 | 0.63 | 1.44 | (0.42, 4.93) | 0.56 | |
| Excess systemic antibiotic use for LRTIs first year of life = Yes | 2.10 | 0.91 | 8.19 | (1.38, 48.75) | 0.02 | |
| Interaction term | 1.74 | 1.07 | - | - | 0.11 | |
| Evaluation of effect modification by parental asthma |  |  |  |  |  | |
| Crude model |  |  |  |  |  | |
| Excessive systemic antibiotic use for parental asthma = No | 0.94 | 0.49 | 2.56 | (0.98, 6.68) | 0.05 | |
| Excessive systemic antibiotic use for parental asthma = Yes | 0.92 | 1.49 | 2.50 | (0.13, 46.77) | 0.54 | |
| Interaction term | -0.02 | 1.57 | - | - | 0.99 | |
| Adjusted model^f^ |  |  |  |  |  | |
| Excessive systemic antibiotic use for parental asthma = No | 1.07 | 0.54 | 2.93 | (1.03, 8.38) | 0.04 | |
| Excessive systemic antibiotic use for parental asthma = Yes | 1.54 | 1.59 | 4.64 | (0.21, 104.48) | 0.33 | |
| Interaction term | 0.46 | 1.65 | - | - | 0.78 | |
| Evaluation of effect modification by atopic dermatitis |  |  |  |  |  | |
| Crude model |  |  |  |  |  | |
| Excessive systemic antibiotic use for atopic dermatitis = No | 1.09 | 0.69 | 2.99 | (0.77, 11.50) | 0.11 | |
| Excessive systemic antibiotic use for atopic dermatitis = Yes | 0.66 | 0.61 | 1.94 | (0.59, 6.36) | 0.27 | |
| Interaction term | -0.43 | 0.92 | - | - | 0.64 | |
| Adjusted model^g^ |  |  |  |  |  | |
| Excessive systemic antibiotic use for atopic dermatitis = No | 1.17 | 0.71 | 3.24 | (0.81, 12.97) | 0.10 | |
| Excessive systemic antibiotic use for atopic dermatitis = Yes | 0.79 | 0.67 | 2.20 | (0.59, 8.24) | 0.24 | |
| Interaction term | -0.39 | 0.94 | - | - | 0.68 | |
| ETS: Environmental tobacco smoke; LRTIs: Lower respiratory tract infections; β: regression coefficient; SE: standard error; IDR: Incidence density ratio; CI: confidence interval; p: p-value; ^a^Adjusted for confounding by sex, parental education, day-care attendance and ETS; ^b^Adjusted for confounding by parental education, day-care attendance and ETS and taking into account effect modification by sex; ^c^Adjusted for confounding by sex, day-care attendance and ETS and taking into account effect modification by parental education; ^d^Adjusted for confounding by sex, parental education and day-care attendance and taking into account effect modification by ETS; ^e^Adjusted for confounding by sex, parental education, day-care attendance and ETS and taking into account effect modification by LRTIs in the first year of life; ^f^ Adjusted for confounding by sex, parental education, day-care attendance and ETS and taking into account effect modification by parental asthma; ^g^ Adjusted for confounding by sex, parental education, day-care attendance and ETS and taking into account effect modification by atopic dermatitis | | | | | | |
